# Registered nurses’ perceptions of food and mealtimes in palliative care: a cross-sectional study

**DOI:** 10.1186/s12904-025-01935-8

**Published:** 2025-11-29

**Authors:** Viktoria Wallin, Andreas Rosenblad, Carina Lundh Hagelin, Anna Klarare

**Affiliations:** 1 Department of Health Care Sciences, Marie Cederschiöld University, Box 11189, Stockholm, 100 61 Sweden; 2 Department of Statistics, Uppsala University, Uppsala, Sweden; 3 Department of Medical Sciences, Division of Clinical Diabetology and Metabolism, Uppsala University, Uppsala, Sweden; 4 Department of Neurobiology, Care Sciences and Society, Division of Family Medicine and Primary Care, Karolinska Institutet, Solna, Sweden; 5 The Connell School of Nursing, Discovery and Implementation for the Common Good, Boston College, Chestnut Hill, Boston, USA; 6 Department of Women’s and Children’s Health, Complex Intervention Research in Health and Care, Uppsala University, Uppsala, Sweden

**Keywords:** Caring, Eating problems, End-of-life, Families, Mealtime, Nursing, Nutrition, Palliative care, Patients, Perceptions

## Abstract

**Background:**

Food and mealtimes are fundamental aspects of human wellbeing, both considering physiological aspects of human life and social interactions. Since registered nurses are key caregivers in palliative care, the aim of this study was to explore registered nurses’ perceptions of food and mealtimes in palliative care.

**Methods:**

An exploratory and descriptive cross-sectional, study-specific survey, designed following a systematic review of the literature, was administered online. The study-specific questionnaire consisted of statements about mealtimes in palliative care, and registered nurses were asked to rate the extent to which they agreed with each statement. Using linear regression analysis associations between socio-demographic variables and registered nurses’ perceptions were explored. Additionally, one open-ended question was analysed using deductive content analysis.

**Results:**

Registered nurses (*n* = 100) had a mean score of 3.3 on the 4-point scale, indicating agreement with the statements about food and mealtimes. Registered nurses agreed to the largest extent with statements concerning r*egistered nurses’ responsibilities* (mean score 3.7), and to the least extent regarding food as *improving health and well-being* (mean score 2.8). Higher age among registered nurses was statistically significant and associated with a lower degree for *food and mealtimes are perceived as distressing* (*P* = 0.004) for patients and family. The open-ended question about “what *advice would you give a new colleague about food and mealtimes in palliative care?”* pertained to the *physical* (56%), *the social* (14%), the *psychological* (9%), and the e*xistential dimensions* (4%), palliative care approach was covered by 17% of the text.

**Conclusions:**

Registered nurses reported that food and mealtimes in palliative care cause distress for patients and families. They strongly agreed on the importance of addressing issues around food and mealtimes at the end of life, helping patients and families to understand that it is common to stop eating as death approaches. Advice to new colleagues focused mainly on physical care, with fewer registered nurses offering guidance on psychological, social, or existential dimensions. This study highlights the need for support in adopting a holistic approach to mealtimes in palliative care.

## Background

The core of nursing entails promoting health, preventing illness, restoring health, and alleviating suffering [1], and is well aligned with palliative care ideals of holistic, person-centred care [2]. A recent integrative review outlines core nursing values of specialist palliative nursing as care, compassion, and commitment [1], emphasizing that registered nurses (RN) are uniquely positioned to enhance patient-led care, to be authentically present, and to make a difference. In a hospital setting, core competencies of specialist palliative care nursing were symptom management and discharge planning, though highlighting that the expectations and the role need further clarification [3]. Patients living with chronic life-limiting diseases, i.e. chronic conditions where death is the likely outcome, have described forcing themselves to eat aiming to improve health and to resist death [4]. Difficulties in eating are related to decreased well-being [5]. Aspects of mealtimes, food preparation and intake, have been described as sources of significant distress for patients [6, 7] and their families [8, 9]. Additionally, as food and mealtimes are linked to memories and identity [10], having eating difficulties fundamentally affect patients’ interactions with friends and family [11, 12].

Palliative care prioritizes the well-being and quality of life of patients throughout their illness trajectory, emphasizing holistic care, interprofessional collaboration and symptom management [2]. During the course of illness, for patients in palliative care, appetite, willingness to eat, everyday eating patterns and participation in mealtimes might shift significantly from those established prior to the onset of disease, particularly in the final stages of life [13]. To adequately address eating-related distress in palliative care, for patients, as well as family members, clinical guidelines should consider holistic, multimodal care, including education and psychosocial support [7, 14]. However, no such guidelines currently exist, resulting in haphazard care related to stressors and eating deficiencies in palliative care [15].

The area of food and mealtimes at the end-of-life has been identified as challenging since difficulties eating disrupt several dimensions, including the social life [9, 16]. Joy around food and mealtimes diminishes and forced eating to please others and to postpone death is prevalent [16]. Patients and families often report on unmet support needs in relation to nutritional care [17]. According to the European Society for Clinical Nutrition and Metabolism (ESPEN) quality of life is the goal of nutritional care and therapy for patients in late phases of end-stage diseases [18]. RNs encounter patients and families with palliative care needs, often independently as RNs provide palliative care aligned with the nursing process [19]. Both patients and families value individual care, social support, and meaningful activities in palliative care [20].

During patients’ illness trajectories, and even close to death, eating difficulties and questions related to food and mealtimes can be of critical importance to patients and families, significantly affecting well-being and daily life. Since RNs work closely to the patients and often are responsible for care planning, exploring RNs’ perspectives on these subjects is, therefore, needed. The aim of the present study was to explore RNs’ perceptions of food and mealtimes in palliative care, including their clinical perspectives of patients and families. An additional aim was to investigate associations between RNs’ socio-demographic characteristics and perceptions of mealtimes.

## Methods

### Study design and context

We designed an exploratory and descriptive cross-sectional survey concerning RNs’ perceptions of food and mealtimes in palliative care (See Table 1). The survey was delivered online via advertised links in two Swedish forums for RNs in palliative care. The STROBE reporting guideline for cross-sectional studies was used to structure the research report [21].

**Table 1:** Statements (*n* = 19) included in the questionnaire and the order of the statements

Item	Statement
1.	Many patients experience mealtimes as distressing at the end of life
2.	Difficulties eating remind patients that death is closing in
3.	Patients who manage to eat despite deficiencies experience increased well-being
4.	Difficulties eating make patients withdraw from family and friends
5.	Food and mealtimes entail community and promote relationships
6.	Patients show their will to live by eating
7.	Patients who manage to eat gain vitality and the energy to live longer
8.	For most patients, the illness and bodily changes make it impossible to eat at the end of life
9.	It is common to stop eating when death is approaching
10.	Patients force themselves to eat for the sake of their families
11.	Conflicts over food and mealtimes between patients and families are common
12.	Family members experience a sense of responsibility for the patient’s eating
13.	Family members who are women take greater responsibility for the patient’s eating than family members who are men
14.	Family members organize food and mealtimes because they want to maintain everyday routines
15.	Family members focus on food because it is a way to keep death away
16.	Family members should accept that patients cannot manage to eat
17.	RNs should help patients accept that it is common to stop eating at the end of life
18.	RNs should help family members accept that it is common to stop eating at the end of life
19.	RNs should take an active part in the work with food and mealtimes at the end of life

Sweden has advanced health system integration of palliative care [22, 23] and right to palliative care irrespective of diagnosis is stipulated in national standards and guidelines [24], In Sweden, palliative care is primarily provided outside hospitals, i.e., in primary health care or specialist homecare [25], though geographic variations and lack of coverage have been found [26]. Palliative care is embedded in both primary and specialized care, and patients can often be cared for at home irrespective of level of care, the own home is oftentimes the most preferred place of end-of-life care and death [27]. Palliative care is included within Swedish undergraduate nursing education to some extent, but this varies between colleges/universities [28, 29]. In 2013, a government commission granted colleges/universities credentials to start specialist palliative care education and training for RNs. According to the Swedish Government’s Official Report [30], 97 RNs had started the program during 2013–2017, but there are no reports of the number of graduated specialist palliative care nurses. Overall, there are approximately 48 000 active RNs in the country.

### Data collection – survey procedure

RNs were recruited by convenience sampling, through a closed group in social media for specialist palliative care RNs, and in a newsletter for a national association for specialist palliative care RNs (approximately 80 and 400 members, respectively). The researchers contacted the administrator of the group in social media and the chair of the association, sharing written information about the study and an invitation to distribute the survey. The survey was distributed via an online link and was available during November and December 2020. Written information about the study and the online link were shared by the administrators. In the social media group, two reminders were posted at two-week intervals. The study invitation included in the newsletter from the national association was distributed via email only once. Responses were gathered via *SurveyMonkey©*, a web-based tool used to collect data via online surveys. The survey was accessible without a need for a password, and due to the anonymous design, IP addresses were not recorded. The order of the items in the survey was fixed, i.e., items were not changed or randomized depending on participant characteristics.

Potential participants were provided written information about the survey by e-mail or via a post in a social media at a group, including a link to the survey. In-depth information was provided on the initial web page when accessing the survey. The information provided was that participation was voluntary and anonymous, and that the collected data were to be analysed on group level and published in a scientific journal. Contact information to the principal investigator, Anna Klarare and first author Viktoria Wallin, was also provided. After the written information, those who wanted to participate were asked to tick a box indicating consent before gaining access to the questions.

The two groups from which members i.e., eligible participants were recruited had a combined total of 480 members, of whom 100 chose to participate in the study. Non-response was discussed with the two individuals responsible for disseminating information about the study, resulting in an estimated response rate of approximately 21%. The survey started with questions concerning socio-demographic data and continued with questionnaire items, using 19 statements with fixed answers, and one open-ended question. Questions related to socio-demographic data and questionnaire items with fixed answers had to be completed to move on to the next item, whereas the open-ended question could be left blank. All proceedings adhered to ethical principles outlined by the World Medical Association [WMA] [31], and the study was approved by the Swedish Ethical Review Authority (number 2020–04618).

### Socio-demographic variables

The following socio-demographic variables were collected: age (year of birth); gender (female/male/not binary/do not want to disclose); number of years since graduation (year of undergraduate RN exam); post-graduate degree in palliative care (Yes/No); other post-graduate degree than palliative care (Yes/No); if “Yes”, i.e., other post-graduate degree, specify which (free text); location of work place (city/suburb/small town/rural); work place (acute care/palliative care or hospice/specialist palliative homecare/primary care/elderly care/other); number of years working as RN (< 1/1–5/6–10/> 10 years); and number of years working in palliative care (< 1/1–5/6–10/> 10 years).

### Questionnaire design

We constructed a study specific questionnaire based on a recent systematic review, which included 24 articles concerning patients’ perspectives of mealtimes in palliative care [32]. We depicted the main findings from the review to explore RNs perceptions of these findings, i.e., (1) Food and mealtimes perceived as distressing, and (2) Affecting social life and interactions, (3) Improving health and well-being as well as (4) Food symbolizing life. Additionally, we included statements about RNs’ perceptions of their responsibilities regarding food and mealtimes in palliative care. These statements, informed by a nursing perspective, are grounded in comprehensive literature searches and reviews within the field of palliative care and nutrition, and are further supported by an empirical study conducted by the first and last authors [33].

Content validity, or how well the survey items represent the subject matter [34], was individually reviewed by 13 people: patients in palliative care (*n* = 2), dieticians (*n* = 3), physicians (*n* = 2), and RNs (*n* = 6). Seven of the clinicians were also active researchers. The written comments were summarized by the first author and subsequently discussed in a workshop with all authors. As a result, two of the statements were clarified and revised (items 16 and 19). The questionnaire was subsequently pilot tested to assess face validity [35], with five RNs working in the context of interest. The questionnaire was found to be clear, relevant, and comprehensible, and thus deemed suitable for the study. No further revisions were made. Please see Table 1 for the statements (*n* = 19). 

In the final version of the questionnaire, based on the literature, four statements were linked to the domain *food and mealtimes perceived as distressing* (items 1, 2, 10, and 11), five to *affecting social life and interactions* (items 4, 5, 12, 13 and 14), three to *improving health and well-being* (items 3, 6, and 7), and four to *food symbolizing life* (items 8, 9, 15 and 16). Eleven items are stated from the patients’ perspectives (items 1–11), whereas five are stated from family members’ perspectives (items 12-16). In addition, three statements concern RNs’ perceptions of their responsibilities regarding food and mealtimes in palliative care: *helping patients/family members to accept that it is common to stop eating at the end of life* (items 17 and 18) and *taking an active part in food and mealtime activities in clinical practice* (item 19).

The RNs rated each item on a 4-point Likert scale: *Strongly disagree* (1 point), *Disagree* (2 points), *Agree* (3 points), and *Strongly agree* (4 points). Mean values were calculated for each item, for the five domains (*distressing*, *affecting social life and interactions*, *improving health and well-being*, *food symbolizing life*, and *RNs’ responsibilities*), as well as for the total 19 items.

The RNs were asked an open-ended question: *Based on your experience*,* what advice would you give a new colleague about food and mealtimes in palliative care?* The open-ended question was voluntary to answer, and RNs could complete the survey whether they responded to this question or not. There were no limits regarding number of letters or words for the question.

### Statistical analyses

Categorical data are presented as frequencies and percentages, n (%), while ordinal and continuous data are given as means and standard deviations (SDs). For ease of presentation, frequencies and percentages for Yes/No variables are only given for the “Yes” group, since the corresponding values for the “No” group are easily inferred. Unadjusted and adjusted linear regression analysis were used to estimate the magnitude of the associations between all included socio-demographic variables: age (years), female gender (Yes/No), years since nurse exam, post-graduate degree in palliative care (Yes/No), post-graduate degree not in palliative care (Yes/No), working in larger urban area or suburb (Yes/No), working in specialist palliative care service (Yes/No), work experience as RN (0–5 years [reference]/6–10 years/> 10 years) and work experience in palliative care (0–5 years [reference]/6–10 years/> 10 years) (predictors) and RNs’ perceptions of food and mealtimes in palliative care (outcome), separately for the five domains as well as the total group. For all Yes/No variables, “No” was used as reference category.

The adjusted analyses included age, female gender and all statistically significant variables from the unadjusted analyses. The results are presented as slope coefficient β with accompanying 95% confidence intervals (CIs). All statistical analyses were performed in R 4.0.0 (R Foundation for Statistical Computing, Vienna, Austria) using two-sided statistical tests with P-values < 0.05 considered statistically significant. No adjustments for multiple comparisons were performed, in line with the arguments provided by Rothman [36] that such adjustments should be avoided when the analysed data are not random numbers but empirical data from actual observations.

### Analysis of the open-ended question

Deductive content analysis [37] was used to categorize responses according to the palliative care dimensions: *physical*,* psychological*,* social*, or *existential* [38]. We created a structured matrix, based on the described competencies related to each dimension in the European Association for Palliative Care (EAPC) position paper, “White Paper on standards and norms for hospice and palliative care in Europe” [39], and relevant data not fitting any of these four dimensions were categorized as *other*. In the next step, these data were analysed based on principles of manifest inductive content analysis [37], resulting in codes such as *person-centred care*, *relief of suffering*, and *family caregivers as central*. From these codes, an overarching category emerged to capture the overall content. NVivo software (QRS International Pty. Ltd., Australia) was used for this analysis, and it was independently validated by the first and last authors.

## Results

### Characteristics of the study sample

Most of the 100 participating RNs (94%) were female, they were on average 48.0 (SD 10.0) years old and had completed their nursing degree a mean (SD) 19.0 (SD 10.4) years ago. Four out of five RNs (81%) worked in specialist palliative care units, 41% had post-graduate degrees in palliative care, and 43% had worked > 10 years in palliative care. Characteristics of participating RNs are presented in Table 2.

**Table 2:** Background characteristics of the participants (*n* = 100)

Variable	Value
Age (years), mean (SD)	48.0 (10.0)
Female gender, n (%)^a^	94 (94.0)
Male gender, n (%)	4 (6.0)
Years since nurse exam, mean (SD)	19.0 (10.4)
Post-graduate degree, palliative care, n (%)^a^	41 (41.0)
Post-graduate degree, not palliative care, n (%)^a^	38 (38.0)
Working in larger urban area or suburb, n (%)^a^	36 (36.0)
Working in specialist palliative care division, n (%)^a^	81 (81.0)
Work experience as RN, n (%)	
– 0–5 years	6 (6.0)
– 6–10 years	19 (19.0)
– > 10 years	75 (75.0)
Work experience in palliative care, n (%)	
– 0–5 years	28 (28.0)
– 6–10 years	29 (29.0)
– > 10 years	43 (43.0)

There were no missing values for any of the variables

*RN* Registered nurse, *SD* Standard deviation

^a^Yes/No variable

### Registered nurses’ perceptions of food and mealtimes in palliative care

On average, RNs agreed to the largest extent to the given statements when these concerned *RNs’ responsibilities regarding food and mealtimes in palliative care* (mean score 3.7 points), while they agreed to the least extent regarding statements concerning *Improving health and well-being* (mean score 2.8 points). The items with the highest scores were: *It is common to stop eating when death is approaching* (3.8 points) and *RNs should help family members accept that it is common to stop eating at the end of life* (3.8 points). The items with the lowest mean scores were: *Patients show their will to live by eating* (2.7 points) and *Patients who manage to eat get vitality and energy to live longer* (2.7 points). Table 3 presents RNs ratings (means and SD) on each statement, for each of the five domains, as well as the total score for the 19 statements.

**Table 3:** RNs’ (*n* = 100) perceptions of food and mealtimes in palliative care

Item	Domains and statements	mean	SD
	*Food and mealtimes perceived as distressing*		
1.	Many patients experience mealtimes as distressing at the end of life	3.3	0.6
2.	Difficulties eating remind patients that death is closing in	3.1	0.6
10.	Patients force themselves to eat for the sake of their families	3.2	0.5
11.	Conflicts over food and mealtimes between patients and families are common	3.5	0.6
	Items 1–2, 10–11	3.3	0.3
	*Affecting social life and interactions*		
4.	Difficulties eating make patients withdraw from family and friends	2.8	0.6
5.	Food and mealtimes entail community and promote relationships	3.5	0.6
12.	Family members experience a sense of responsibility for the patient’s eating	3.6	0.6
13.	Family members who are women take greater responsibility for the patient’s eating than family members who are men	2.9	0.8
14.	Family members organize food and mealtimes because they want to maintain everyday routines	3.1	0.7
	Items 4–5, 12–14	3.2	0.4
	*Improving health and well-being*		
3.	Patients who manage to eat despite deficiencies experience increased well-being	2.9	0.7
6.	Patients show their will to live by eating	2.7	0.7
7.	Patients who manage to eat gain vitality and the energy to live longer	2.7	0.7
	Items 3, 6–7	2.8	0.5
	*Food symbolizing life*		
8.	For most patients, the illness and bodily changes make it impossible to eat at the end of life	3.4	0.8
9.	It is common to stop eating when death is approaching	3.8	0.5
15.	Family members focus on food because it is a way to keep death away	3.3	0.6
16.	Family members should accept that patients cannot manage to eat	3.3	0.6
	Items 8–9, 15–16	3.5	0.4
	*RNs’ responsibilities*		
17.	RNs should help patients accept that it is common to stop eating at the end of life	3.6	0.5
18.	RNs should help family members accept that it is common to stop eating at the end of life	3.8	0.4
19.	RNs should take an active part in the work with food and mealtimes at the end of life	3.6	0.6
	Items 17–19	3.7	0.4
	*Total*		
	Items 1–19	3.3	0.3

There were no missing values for any of the variables

*RN* Registered nurse, *SD* Standard deviation

### Associations between socio-demographic variables and registered nurses’ perceptions

Results of the linear regression analysis of the associations between age (years), female gender, post-graduate degree, outside palliative care, and work experience as RN (predictors) and RNs’ perceptions of food and mealtimes in palliative care (outcome) are shown in Table 4.

**Table 4:** Results of linear regression analysis of the associations between socio-demographic variables (predictors) and RNs’ perceptions of food and mealtimes in palliative care (outcome)

		Unadjusted		Adjusted^a^	
Domain	Predictor	β (95% CI)	*P*-value	β (95% CI)	*P*-value
Distressing	Age (years)	−0.006 (−0.013; 0.001)	0.111	−0.012 (−0.020; −0.004)	**0.004**
	Female gender^b^	0.287 (−0.0005; 0.575)	0.0504	0.271 (0.002; 0.541)	**0.049**
	Post-graduate degree, not in palliative care^b^	−0.181 (−0.320; −0.042)	**0.011**	−0.185 (−0.317; −0.053)	**0.007**
	Working experience as RN				
	– 0–5 years	Reference			
	– 6–10 years	−0.311 (−0.630; 0.007)	0.055	−0.213 (−0.510; 0.083)	0.156
	– > 10 years	−0.117 (−0.405; 0.172)	0.424	0.137 (−0.157; 0.431)	0.358
Affecting social life and interactions	Age (years)	−0.002 (−0.010; 0.006)	0.644	−0.008 (−0.018; 0.002)	0.109
	Female gender^b^	0.389 (0.047; 0.732)	**0.026**	0.342 (−0.001; 0.685)	0.051
	Post-graduate degree, not in palliative care^b^	0.072 (−0.099; 0.243)	0.407	0.072 (−0.096; 0.241)	0.395
	Working experience as RN				
	– 0–5 years	Reference			
	– 6–10 years	−0.475 (−0.852; −0.098)	**0.014**	−0.392 (−0.770; −0.015)	**0.042**
	– > 10 years	−0.236 (−0.578; 0.106)	0.173	−0.110 (−0.484; 0.265)	0.562
Improves health and well-being	Age (years)	0.004 (−0.007; 0.014)	0.506	−0.001 (−0.015; 0.012)	0.875
	Female gender^b^	0.058 (−0.394; 0.510)	0.800	−0.027 (−0.493; 0.438)	0.908
	Post-graduate degree, not in palliative care^b^	0.007 (−0.215; 0.228)	0.954	−0.029 (−0.257; 0.200)	0.805
	Working experience as RN				
	– 0–5 years	Reference			
	– 6–10 years	−0.272 (−0.768; 0.224)	0.279	−0.271 (−0.783; 0.242)	0.297
	– > 10 years	−0.007 (−0.456; 0.442)	0.977	0.018 (−0.491; 0.526)	0.945
Prolongs life	Age (years)	−0.003 (−0.010; 0.005)	0.489	−0.006 (−0.015; 0.003)	0.224
	Female gender^b^	0.090 (−0.211; 0.392)	0.554	0.102 (−0.210; 0.414)	0.517
	Post-graduate degree, not in palliative care^b^	−0.084 (−0.231; 0.063)	0.259	−0.087 (−0.240; 0.066)	0.263
	Working experience as RN				
	– 0–5 years	Reference			
	– 6–10 years	0.004 (−0.333; 0.342)	0.979	0.047 (−0.296; 0.389)	0.788
	– > 10 years	0.057 (−0.249; 0.362)	0.714	0.175 (0.165; 0.516)	0.308
RNs’ responsibilities	Age (years)	−0.003 (−0.011; 0.004)	0.400	−0.001 (−0.011; 0.009)	0.823
	Female gender^b^	0.025 (−0.294; 0.344)	0.878	0.043 (−0.289; 0.376)	0.797
	Post-graduate degree, not in palliative care^b^	−0.038 (−0.194; 0.118)	0.633	−0.017 (−0.181; 0.146)	0.832
	Working experience as RN				
	– 0–5 years	Reference			
	– 6–10 years	−0.096 (−0.451; 0.258)	0.590	−0.085 (−0.450; 0.281)	0.647
	– > 10 years	−0.167 (−0.487; 0.154)	0.305	−0.142 (−0.505; 0.221)	0.438
Total	Age (years)	−0.002 (−0.007; 0.003)	0.407	−0.006 (−0.012; 0.00003)	0.051
	Female gender^b^	0.195 (−0.018; 0.408)	0.072	0.171 (−0.041; 0.383)	0.113
	Post-graduate degree, not in palliative care^b^	−0.042 (−0.147; 0.064)	0.434	−0.045 (−0.150; 0.059)	0.389
	Working experience as RN				
	– 0–5 years	Reference			
	– 6–10 years	−0.248 (−0.482; −0.014)	**0.038**	−0.194 (−0.428; 0.039)	0.102
	– > 10 years	−0.102 (−0.314; 0.110)	0.342	0.017 (−0.214; 0.249)	0.884

None of the variables Years since nursing exam; Post-graduate degree, in palliative care; working in larger urban area or suburb; Working in specialist palliative care division; or Working experience in palliative care were statistically significant in any of the unadjusted analyses

*CI* Confidence interval, *RN* Registered nurse

^a^Adjusted for all other variables in the domain or total.

^b^Yes/No variable, with “No” used as reference category

Significant *P*-values are given in bold

In the adjusted analyses, female gender was significantly associated with higher agreements on statements that *food and mealtimes are perceived as distressing* (*P* = 0.049) and borderline significant (*P* = 0.051) for *food and mealtimes affect social life and interactions* (*P* = 0.026). Higher age was significantly associated with a lower degree of agreement on statements that *food and mealtimes are perceived as distressing* (*P* = 0.004). Moreover, having a post-graduate degree in an area other than palliative care was significantly associated with lower agreements on statements that *food and mealtimes are perceived as distressing* (*P* = 0.007), while having a 6–10 years’ work experience as RN implied lower agreement on statements that *food and mealtimes affect social life and interactions*, compared to RNs with 0–5 years work experience (*P* = 0.042).

On average, females had a 0.271 points higher agreement on *food and mealtimes are perceived as distressing*, and a 0.342 points higher agreement for statements in the domain *affect social life and interactions*. RNs perceived that food, and mealtimes were on average 0.012 points less distressing for each additional year old they were, while RNs with a post-graduate degree in an area other than palliative care had a 0.185 points lower agreement with the statement that *food and mealtimes are perceived as distressing*. Finally, RNs with a work experience as RN of 6–10 years on average had a 0.392 points lower agreement on statements that *food and mealtimes affect social life and interactions*, compared with RNs having a shorter work experience of 0–5 years.

### Registered nurses’ advice to colleagues

Most RNs (90%) answered the open-ended question, and the responses collected ranged from two to 330 words (mean 32 words). The total word count from all responses was 3197 words. Responses to the open-ended question regarding advice to a new colleague mostly pertained to physical dimensions of care, followed by psychological, social, and existential dimensions (Table 5).

**Table 5:** RNs’ (*n* = 90) advice to new colleagues sorted according to WHO’s palliative care dimensions and other dimensions, numbers of RNs given answers belonging to each dimension, number of codes, and percentage of text belonging to each dimension

Dimension		Number of RNs	Number of codes	Percentage of text
Palliative care dimensions	Physical	61	69	56
	Social	25	29	14
	Psychological	20	21	9
	Existential	7	9	4
Other	Palliative care approach	10	20	17

The advice regarding the *physical dimension*, described by 61 RNs, included 69 codes that focused on aspects of eating and how to facilitate and maintain eating throughout the illness trajectory. Examples included practical advice such as adjusting size of meals and food options and eliminating physical reasons for inability to eat (ulcers, mycosis, strictures, etc.). The *social dimension*, described by 25 RNs, included 29 codes that pertained to advising new colleagues about family interventions, e.g., that family members should be given space to eat together with patients. Responses also highlighted RNs’ responsibilities to inform and educate families regarding the dying process and reduced food intake. The most common responses covered individual support to family members and that RNs should help families find strategies to navigate food and eating towards the end-of-life. This could be achieved if families could understand physical deterioration during the dying process and that food no longer is necessary. Advice concerning the *psychological dimension*, described by 20 RNs, included 21 codes regarding advice to the new colleagues about aspects of enjoyment around food. Several RNs underscored that eating should never be forced and that nagging was negative. Advice regarding the *existential dimension*, described by seven RNs, included nine codes that focused on life experiences and meanings of eating, like keeping death at bay.

A majority (56%) of the responses to the open-ended question pertained advice regarding the *physical dimension.* In contrast, 14% and 9% of the advice pertained to *the social* and *psychological dimensions*, respectively. *Existential dimensions* were covered by 4%, and palliative care approach in 17% of the text.

In the *other category*, advice from 10 RNs to new colleagues (20 codes) was given concerning strategies for nursing and how to achieve a palliative care approach. The most common advice was to focus on patients’ needs and wishes to form care interventions, accordingly, emphasizing that *person-centred care*, *relief of suffering*, and *family caregivers are central*. Using attributes such as humility and respect was also encouraged.

To summarize the qualitative data, the physical dimension pertained to practical advice, such as adjusting size of meals and food options, and eliminating physical reasons for inability to eat. The psychological dimension pertained to reactions and emotions such as frustration or sadness. The social dimension pertained to social interactions such as not eating together or not eating the same food and the existential dimension pertained to thoughts of starvation and food as life sustaining. Data not related to these dimensions comprised descriptions concerning the palliative care approach.

## Discussion

This study presents an initial attempt to study RNs’ perceptions about food and mealtimes in relation to specialist palliative care. The RNs in this study agreed that food and mealtimes in palliative care causes psycho-social distress for patients and families. Female gender was significantly associated with higher agreements on statements that *food and mealtimes are perceived as distressing* and *affect social life and interactions*, as well as with overall higher agreements on the 19 statements. On the other hand, higher age among RNs was associated with a lower degree of agreement that *food and mealtimes are perceived as distressing*. Furthermore, RNs strongly agreed that they should actively work with food and mealtimes at the end of life in *helping patients and families to accept that it is common to stop eating when death is near*. Advice to new colleagues was dominated by physical aspects of care. Fewer RNs suggested advice related to care dimensions with psychological, social, or existential connotations. Support in embracing a palliative care approach was encouraged in general terms.

### Results in perspective

Palliative care philosophy and practice guidelines do emphasize the multidimensional nature of palliative care, comprising physical, psychological, social, and existential needs [2, 38, 39], and a holistic approach to care has been advocated since the early days of the hospice movement [40]. Nonetheless, our results show that operationalizing a holistic approach in palliative care nursing seems to present a challenge. The RNs in this study were experienced, well-educated clinicians and may thus represent a normative care culture with regards to food and mealtimes in palliative care. In other words, chances are that new colleagues are socialized into the same norm, potentially focusing on physical aspects of care and consequently neglecting, either consciously or by omission, psychological, social, and existential aspects of care. A recent meta-analysis of RCTs underscores that integrated, holistic palliative care improves patients’ abilities to cope with physical and psychological burdens of cancer, thus contributing to improved overall well-being [41]. A recent meta-synthesis of coping strategies for patients with uncurable illness, confirms that holistic care is crucial [35]. There is ample evidence to suggest that education and training programs that emphasize the importance of psychological, social, and existential needs in parallel with physical needs is called for. We suggest that these programs should include practical strategies for integrating these aspects into daily care practices of individual RNs, for example as a fellowship or trainee year with mentoring from specialist palliative care RNs [42], further ethical considerations related to mealtimes should be addressed to support person-centred, holistic approaches in palliative care [43].

With the paradigm shift in palliative care to a broader definition [2, 4], subsequently, the scope of specialist nursing practice has shifted from symptom management and death, approaching to include promoting well-being and strategies for living with a chronic illness. Our result indicates this shift may not yet have influenced clinical practice regarding food and nutrition in palliative care. Mealtimes go beyond food intake and physiological needs, and as previously suggested, care should also encompass other dimensions [44, 45]. Existing guidelines primarily focus on physiological needs [46, 47]. Policy changes are needed to directly support holistic care practices. We advocate for the inclusion of comprehensive guidelines on nutritional care in palliative care standards, ensuring that multiple dimensions of patient well-being are addressed.

Reviews indicate that RNs are aware of the illness trajectory, with dying and death affecting patients, families, and care providers [1, 48]. In our results, higher age among RNs was associated with a lower degree of agreement that *food and mealtimes are perceived as distressing*. Similarly, age was found to affect nursing students’ attitudes towards care of the dying [49]. It is unclear whether this is connected to clinical experiences and subsequent personal reflections concerning death and dying or other factors. Benner’s [50] novice to expert theory provides a useful framework for understanding the progression of nursing proficiency and its’ impact on holistic nursing practices. Already when patients are admitted to palliative care, RNs know that dying and death are to be expected, even though patients and families may not yet have arrived at the same conclusion [51]. RNs are expected to support and inform patients and families, with an imperative to help prepare them for a good death. With the diverging understanding and expectations on goals of care between RNs and patients and families, RNs may experience distress since caring for patients and families who are not fully aware of dying is challenging, especially if there are differences or lack of joint goals of care in the healthcare team [52]. In the context of palliative care, Benner’s framework [50] may help explain why experienced RNs tend to prioritize physical aspects of care. In this study, RNs may have focused on physical aspects in relation to food and mealtimes, since there is a multitude of concrete care interventions to try, as suggested by Gillespie and Raftery [53]. Their advanced clinical skills allow them to address concrete needs efficiently, whereas e psychological, social, and existential needs may require more nuanced and intuitive approaches that develop over time. In outcomes focused healthcare settings, ‘doing’ nursing interventions that ‘solve’ a physical problem may be preferable to something that takes more time and effort. Research suggests that existential needs may be especially challenging since dying and death is a reality in the near future [54]. To be prepared to address existential concerns often involves deep personal reflections on the meaning of life, mortality, and the emotional and spiritual distress associated with the end of life. Nonetheless, provision of specialist palliative care nursing requires engaging with patients and families in creating sensitive, person-centred, and individualized support [2, 48]. Education and training for individual RNs to operationalize holistic care and developing policy guidelines for nutritional care in palliative care are suggestions to fill the gaps identified by this study.

### Strengths and limitations

Constructing a study-specific questionnaire may present a limitation with regards to internal validity [55]. However, statements originated with existing literature and the questionnaire was discussed, tested, and reflected on by a multi-professional group and an expert group for its content and face validity before being used, crucial when constructing a questionnaire [56, 57]. Constructing statements based on research is complex and entails a risk, as they may reflect underlying assumptions made by the researcher. Therefore, testing the statements was essential. Desirability bias [58] is a risk when distributing a questionnaire indicating that there are challenges around food and mealtime. Potentially, RNs may have agreed to participate on those premises, consequently resulting in skewed responses. On the other hand, RNs answered the questions anonymously and could utilize the free text answers for elaboration without any direct influence by researchers. The open-ended question provided RNs with the opportunity to add experiences that were not highlighted in the questionnaire.

Given the low response rate (approximately 21%), no claims of generalization can be made. Nonetheless, 100 persons responded during an ongoing pandemic. Recruiting participants via social media and an association for RNs in palliative care mean that reasons for declining participation were unknown. The two groups might have some overlap, as RNs could potentially be members of both groups. Sample bias is possible due to the convenience sampling; however, some significant results were found, and the results can potentially generate hypotheses for future research. Our sample consisted of RNs with extensive clinical experience, and they were highly educated; many had a post-graduate degree in nursing and were experienced in palliative care, thus further limiting the generalization of the findings. However, the sample potentially does indicate how RNs with specialist education view circumstances around food and mealtimes at the end of life. Initial exploration implies that RNs’ perceptions align with patients’ and families’, indicating awareness of the challenges that patients and families face.

Another aspect to consider is the gender imbalance with 94% of the participants being women. This is somewhat higher than the proportion of female RNs in Sweden at 87% [59]. Despite this gender imbalance, statistically significant gender differences were found, with females to a higher degree agreeing that food and mealtimes are perceived as distressing, and that they affect social life and interactions. Although no conclusions can be drawn regarding the clinical importance of these findings based on our study, the results indicate that exploring these differences further may provide gendered nuances of caring practices. A larger sample focusing on whether gender is associated with perceptions of food and mealtimes, or other core aspects of caring, may yield valuable insights. A larger sample may also have detected potential differences between general and specialised palliative care.

## Conclusions

The RNs in this study reported that food and mealtimes in palliative care cause distress for patients and families. They strongly agreed on the importance of actively addressing issues around food and mealtimes at the end of life, helping patients and families to understand that it is common to stop eating as death approaches. Advice to new colleagues primarily focused on physical aspects of care; with fewer RNs offering guidance on psychological, social, or existential dimensions. Support in embracing a palliative care approach was encouraged in general terms, aligning with professional competence and development. This study highlights the need for support in adopting a holistic approach to mealtimes in palliative care.

## Data Availability

The questionnaire statements are included in the article. The datasets used and/or analysed during the current study are available from the corresponding author upon reasonable request.
